# Editorial: Revolutionizing life sciences: the nobel leap in artificial intelligence-driven biomodeling

**DOI:** 10.3389/fmolb.2024.1540823

**Published:** 2025-01-03

**Authors:** Valentina Tozzini, Cecilia Giulivi

**Affiliations:** ^1^ Istituto Nanoscienze del Consiglio Nazionale delle Ricerche (CNR), Lab NEST-Scuola Normale Superiore, Pisa, Italy; ^2^ Istituto Nazionale di Fisica Nucleare (INFN), Sezione di Pisa, Pisa, Italy; ^3^ Department of Molecular Biosciences, School of Veterinary Medicine, University of California Davis, Davis, CA, United States; ^4^ MIND Institute, University of California at Davis Medical Center, Sacramento, CA, United States

**Keywords:** deep-learning, neural networks, structure prediction, drug design, disordered proteins, biomolecules interactions

## 1 Artificial intelligence’s impact on biomolecular modeling

Within the research world, 2024 will be remembered as the year of Nobel Prizes for Artificial Intelligence (AI). The one for Physics, awarded to John Hopfield and Geoffrey Hinton *for foundational discoveries and inventions that enable machine learning with artificial neural networks*, has sealed the connection between physics and information science, now officially mating on a strongly interdisciplinary frontier field after over 50 years of fruitful interaction (Artificial, 2024). More specifically, connecting AI to biomolecular modeling relates to the Nobel Prize in Chemistry awarded to David Baker *for computational protein design* and to Demis Hassabis and John Jumper *for protein structure prediction*.

**FIGURE 1:**
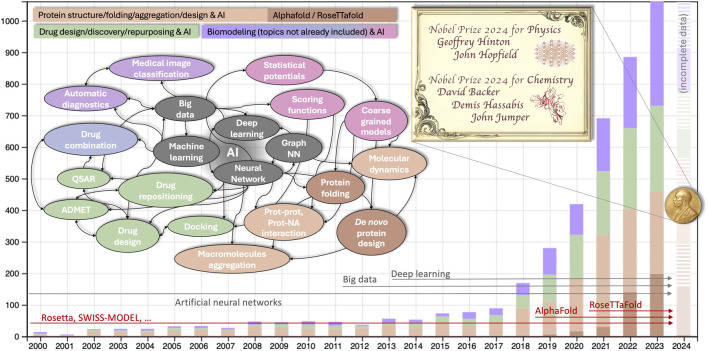
Number of publications on machine learning in biological modeling and simulation from 2000-present. The search was performed using the keywords (computer modeling OR simulation) AND (machine OR deep OR automatic learning OR neural networks) AND (proteins OR nucleic acids OR biomolecules) either in the full text (∼120K items since 2000) or only in the abstract (∼6,000, analyzed and shown data) both in Scopus and WoS database (shown data are from WoS, 2024 incomplete). Colors of the histograms are described in the legend (purple is for generic bio-modeling not already included in the drug or protein design, in green and orange respectively). The colors in the conceptual map correspond to that of the histogram, with additional shades of purple for different generic biomodelling tasks other than protein or drug design. Horizontal arrows illustrate when the main keywords related to AI (gray) and to AI-based protein modeling (red) become statistically relevant in the literature.

Numerous statistics illustrate the influence of artificial intelligence in the field of biomodeling. An inquiry conducted in scientific literature databases employing AI-related keywords pertinent to the computer modeling of biomolecules yields approximately 120,000 results (approximately 6,000 results if the search is confined to the abstract, as illustrated in Figure 1). The exponential rise observed starting from 2018–19 was the prelude to the Nobel, and approximately coincides with the appearance of the two software suites, AlphaFold (Senior et al., 2019) and RosettaFold (Humphreys et al., 2021), which implement the methods for proteins folding and proteins *de novo* design developed by Hassabis/Jumper and Baker, respectively.

Receiving a Nobel Prize just a few years after the awarded research is quite rare, but certainly not accidental. The methods for protein structure prediction based on homology modeling were developed starting in the 1990s and implemented in popular software suites, including the early version of Rosetta (Bowers et al., 2000) and others [e.g., SWISS-MODEL (Guex and Peitsch, 1997)]. These methods heavily depend on statistical data. They involve aligning and ranking sequences and structures and parameterizing scoring functions through extensive analysis of sequence and structure databases. This process culminates in distilling the information into a few optimal structures or interaction models (Wang et al., 2019). Over the years, the growing volume of statistical data has necessitated the automation of tasks, particularly in searching and comparing information. Advancements in hardware architecture and storage capacity have supported this shift.

Meanwhile, automatically trained neural networks (NN) have emerged as a natural solution for the “distillation” of this data (Kanada et al., 2024). During the second decade of 2000s, the co-evolution of computer performance and algorithms led to the transition from *machine learning* (ML) to *deep learning* (DL). This shift involved adding layers to the neural networks, resulting in qualitative and quantitative predictive power improvements. The combination of an established supportive environment, the availability of big data, and the rise of DL has significantly contributed to the success of AI methods in bio-modeling.

Specifically regarding protein structure, AlphaFold now achieves an impressive 99% accuracy in predicting single-chain proteins, rendering the CASP challenge—historically focused on structure prediction—less relevant.

Besides the modeling of protein structures, a significant domain of artificial intelligence application elucidated by statistical analysis pertains to drug development. In particular, ML is used to address structure-activity relationships (Gupta et al., 2021) and uptake-toxicity of the drug (De Carlo et al., 2024), virtual screening, and structure-based design. While not claiming to cover all potential applications, we note that optimizing force fields for low-resolution models of biomolecules significantly benefits from machine learning (Kanada et al., 2024; Majewski et al., 2023; Mirarchi et al., 2024), whereas the application of graph neural networks for calculating molecular dynamical trajectories is a cutting-edge approach (Husic et al., 2020).

## 2 AI’s impact on biological modeling and simulation in Frontiers in Molecular Biosciences

Frontiers in Molecular Biosciences (FMB) has witnessed an exponential rise of publications with the exact timing and similar topical distribution, currently counting several hundreds of publications on AI related topics. The section of Biological Modeling and Simulation (BMS) is one the most involved, having issued several Research Topic Collections (Research Topics, RT) on the diverse applications of neural networks in biomolecular simulations, on the prediction of protein structure and conformation, or focusing on data-driven applications, on drug design, even combined with molecular studies of metabolic pathways also in relation to the cancer treatment.

A deeper look into the BMS section also reveals more specific topics out of the mainstream, such as the prediction of protein-protein interactions and the study of the conformation of intrinsically disordered proteins. Indeed, these are two aspects where ML algorithms show their weakness (Abramson et al., 2024), displaying decreased accuracy. This is attributed to the under-representation within the training dataset of crucial features, such as the conformational variability of disordered proteins and protein-protein interfaces (Saldano et al., 2022), especially when combined with sequence variability, e.g., in the study of antibodies (Yin et al., 2022). The decreased accuracy and predictive power in cases “too far” from those included in the learning dataset is considered one of the main drawbacks of automatic learning-based methods.

### 2.1 Beyond the stream and into the niches of AI applications

To explore unconventional AI methods for bio-modeling and showcase niche applications and challenging or problematic areas, we have compiled 15 “orphan” papers in this Research Topic. These papers, which are not part of any existing topical collection, have been published in the sections of Biological Modeling and Simulation or Structural Biology of FMB.

In the review by Zhang et al. it is noted that AlphaFold, along with other similar AI methods for structure prediction, such as RoseTTaFold and EMSFold, is widely used in various fields of biomedical research. In addition to drug design, the authors highlight its applications in immunology, particularly in predicting and designing immunoglobulin structures or developing structure-based vaccines. The work also emphasizes the development of biomarkers, the study of protein-protein and protein-nucleic acid interactions, and the investigation of missense mutations. However, the review points out some limitations of these methods, specifically the decreased accuracy in predicting the relative positioning of large protein domains and their intrinsically disordered regions and challenges in differentiating between various environmental conditions. In this regard, alternative approaches like AminoBERT, described in Zhang et al., demonstrate better performance in *de novo* design or when few homologous sequences are available. This improvement is achieved by omitting the multiple sequence alignment step and instead incorporating residue-based chemical and geometric information.

The absence of specific protein information in the training data and the resulting bias towards the included proteins are two sides of the same coin, which makes the neural network predictions contingent on the dataset’s composition. Sala et al. transformed the challenge into an opportunity by introducing a controlled bias in AlphaFold2 toward specific user-defined subsets of structures. This can be achieved by incorporating genetic information to enhance accuracy for particular protein families. The algorithm has demonstrated improved performance on CPCRs and kinase protein families, which are notably difficult due to their multiple active conformations. Additionally, the capability of AlphaFold to address different or multiple structures was discussed in the mini-review by Hunter et al. This study focused on examining the structure of ALAS synthase, specifically highlighting a predicted divergence in the *C*-terminal domain of the protein and its connection to the proposed allosteric regulation of protein activity.

### 2.2 Integrating AI and simulation techniques: advancing biomolecular structure prediction and drug discovery

Utilizing a diverse array of methods has demonstrated remarkable effectiveness in accurately predicting the structures of biomolecules. The structure predicted by AlphaFold, along with Molecular Dynamics (MD) simulations, served as the reference for evolutionary studies. Just to cite a few ones highlighting this link, the study by Bug et al. on the ribonuclease Dicer1 involved in miRNA biogenesis and hematological cancers progression, and that by Meller et al. to generate the structure of the unknown protein PPM1D phosphatase, an important marker in oncology involved in the regulation of DNA damage response. In these cases, the structure was combined with a graph convolutional network model trained over activity data, and with MD simulations to enhance the drug docking task, revealing an allosteric “cryptic” pocked, not immediately accessible and therefore escaping the structural-only analysis. Belviso et al. used Alphafold and MD in combination with small-angle X-ray scattering to characterize the *C*-terminal region of NSD3 histone lysine methyltransferases, a marker in oncogenesis, showing that combined modeling techniques can be used to augment the low resolution experimental structural characterization techniques.

### 2.3 Advancing drug discovery: integrating AI, simulations, and experimental methods for targeted therapeutics

Drug design increasingly benefits from interdisciplinary approaches combining advanced computational techniquesand ML with experimental validation to accelerate therapeutic discovery and innovation. Zeng et al. used a cascade of structure-based drug design methods combining MD and metadynamics of the drug-target complex with ML-based virtual screening and QSAR and ADMET evaluation. Combined with experimental procedures, this approach identified inhibitors of fibroblast growth factor receptors that were also tumor suppressors.

Drug design represents a promising frontier for advancing NN development, particularly at the algorithmic level. The complexity of molecular interactions, coupled with the need to predict binding affinities, toxicity, and pharmacokinetics, provides a fertile ground for refining and innovating NN architectures. Emerging techniques, such as graph-based neural networks and attention mechanisms, are poised to address these challenges by enabling more accurate modeling of molecular properties and interactions, paving the way for breakthroughs in computational drug discovery. Ni et al. developed a model of a Graph Convolutional Network with a layer attention mechanism and trained it to predict the association of small molecules to target miRNA. Despite the large number of hidden layers and advanced mechanisms to cope with data redundancies and reduce the noise, the authors claim dissatisfaction with the specific task, possibly due to insufficient variability in the dataset. Wu et al. combined an NN with docking and virtual screening to repurpose drugs for Alzheimer’s disease, which allows the optimization of a multi-target approach capable of identifying the network of proteins interacting with the receptor S1R, considered as the starting target, and subsequently identifying several leads, tested by docking and ADMET prediction. To a similar scope of finding effective combinations of drugs for multifactorial diseases, Hong et al. develop a different NN approach independent of structures and based on the Pathway Interaction Network (PINet), which was tested on acute myeloid leukemia, where it correctly predicted midostaurin and gemtuzumab as effective drug combinations and proved particularly effective when the training dataset is limited.

We should pay attention to the early research on antivirals targeting the main protease of SARS-CoV-2 in the context of structure-based drug design. Lau et al. combined molecular docking and MD with a convolutional neural network and spatial graph model trained on ligand-protein data, used to predict the ligand-protein score and identify from a library of 26 million molecules possible candidate compounds to target RBD domain of the Spike protein or Mpro. Using biolayer interferometry for the spike protein and a FRET-based reporter, their effective binding was tested. Samad et al. considered as the target the chymotrypsin-like protease (3CL^PRO^) and used machine learning-based virtual screening of 4,000 phytochemicals. The Random Forest model, displaying 98% accuracy on the train and test set, identified several molecules that were subsequently docked into the target and analyzed by MD. The procedure identified 26 potential inhibitors.

Finally, we mention a couple of applications within the biological modeling area that are out of the mainstream, not on molecular modeling but on using images for diagnostics. Bigler et al. use a deep learning approach with transfer learning of a pre-trained convolutional neural network to identify pathological patterns in skeletal muscle biopsies, using transmission electron microscopy images showing that the learned network is proven superior in the classification concerning commonly used morphometric analyses. More specifically, Qi et al. trained an NN to automatically diagnose suppurative otitis media and middle ear cholesteatoma, proving a handy tool to help physicians discern these two chronic diseases displaying similar CT medical images.

## 3 Perspectives

In the last decade, AI has produced a massive acceleration in biomolecular modeling, making several tasks previously requiring a long time and specific expertise fast and easy. These are, in particular, those involving analyzing and synthesizing information from large amounts of data. The case of AlphaFold is an exemplar: the current version allows even nonexperts in the field to have a prediction of the fold of a protein from the sequence in minutes, a task which required weeks with the traditional homology modeling procedure, and reaching comparable or superior accuracy in most of the cases.

Despite its remarkable progress, AI-driven biomolecular modeling faces significant challenges highlighting the need for caution and critical evaluation. One major issue lies in the bias and incompleteness of training databases. This risks to produce results that reflect the limitations or skewed composition of the input data, potentially leading to inaccurate predictions and amplifies the risk of “hallucinations” – outputs that are highly ranked, but scientifically invalid–possibly due to overfitting and extrapolation beyond known data. Beyond hallucinations, we already commented on the cases of disordered structures and inter-domain interface prediction, whose low confidence the ML models can autonomously evaluate. In addition, AI-driven platforms like DeepMind’s AlphaFold have predicted novel drug candidates for various diseases, but still, several of these compounds need to be sufficiently followed up regarding their pharmacokinetics, such as IC50 values (the concentration needed to inhibit 50% of a target) or their ability to be administered effectively. In some cases, promising compounds identified by AI have yet to pass crucial stages in drug development, such as formulation stability, bioavailability, or FDA approval. A notable case is the identification of AI-generated inhibitors for the SARS-CoV-2 virus, which, while initially promising, failed to meet the necessary clinical standards and were ultimately not pursued for broader therapeutic use.

Furthermore, the need for explainability in many AI models compounds these challenges. Without transparent mechanisms to trace how predictions are made, it becomes difficult for researchers to assess their reliability or identify potential errors. This opacity raises concerns about the reproducibility and trustworthiness of AI-generated insights, particularly in high-stakes fields like drug discovery or biomolecular engineering. Adding explainability to the method, and not only in the biomodelling field, is currently one of the main challenges for developing automatic learning algorithms. On the technical level, one way to address this problem as far as that of (explicit or not) low reliability and bias, is to reduce the complete automatism by re-introducing into the procedure elements of symbolic artificial intelligence based on deductive rules into a hybrid approach known as neuro-symbolic AI (Bhuyan et al., 2024).

On a philosophical level, the growing reliance on AI may inadvertently foster excessive trust in its outputs, sometimes at the expense of scientific scrutiny. This overconfidence could lead to a diminished critical sense, where the technology's predictions are only accepted without adequate validation. For instance, some AI-predicted compounds have led to follow-up studies that overlook crucial aspects like side effects, toxicity, or long-term efficacy, which must be fully captured in the initial models. To mitigate these risks, fostering interdisciplinary collaboration, emphasizing data quality, and developing interpretable AI systems are essential to ensure that AI remains a robust and reliable tool for advancing biomolecular research.

In conclusion, while it is true that AI presents challenges and risks, it also offers transformative opportunities when wielded responsibly. We are at a juncture where AI is no longer just an optional tool but a cornerstone of modern modeling and problem-solving. Like any tool, its effectiveness depends on the skill and wisdom of its user. By combining the power of AI with the irreplaceable intuition and common sense of human judgment, we can harness its potential for innovation and progress, ensuring a future where technology enhances, rather than replaces, our humanity.
